# Integrative Medicine in Diagnostics: Current Advances and Future Prospects

**DOI:** 10.3390/diagnostics13193052

**Published:** 2023-09-25

**Authors:** Bo-Young Youn

**Affiliations:** Department of Bio-Healthcare, Hwasung Medi-Science University, Hwaseong-si 18274, Gyeonggi-do, Republic of Korea; jamesyoun@hsmu.ac.kr

Integrative medicine (IM) has recently gained significant attention from patients and healthcare professionals worldwide. IM is an emerging field that focuses on patient-centered care for preventing, managing, and treating various diseases [1]. Generally, IM integrates evidence-based conventional approaches and complementary therapies to treat the patient’s whole aspects, such as physical, mental, and spiritual needs [2].

This Special Issue aims to highlight new diagnostic approaches in the field of IM that could play vital future roles in delivering more precise diagnoses for the betterment of patient care. A total of ten articles were published in this Special Issue; five from South Korea, two from Iraq, and one each from Japan, Italy, and India.

Amid the published studies, Jang et al. prepared lumbar spine images to undertake a reliability analysis of vertebral landmark labelling [3]. In order to accomplish this objective, 12 manual medicine experts were involved as raters to conduct a reliable analysis of landmark points and vertebral endplate lines. A standard operating procedure was generated by the experts; as a result, high intra-class correlation coefficients ranging from 0.934 to 0.991 verified the reliability.

Park et al. examined the usefulness of Sasang typology, known as a type of Korean medicine, in diagnosis [4]. This study included Sasang-type diagnoses of the 395 healthy participants for distinguishing the mechanism of Tae-Eum-type-specific obesity utilizing the participants’ physical characteristics while enhancing the Tae-Eum diagnosis. As a result, the Tae-Eum-type group demonstrated higher body weight, body fat mass, body mass index, and unstandardized resting metabolic rate (kcal/day) than others. The logistic regression model also revealed that the increased resting metabolic rate is essential for distinguishing the Tae-Eum type as well as exemplifying the specific mechanism of Tae-Eum-type obesity.

Yamamoto et al. investigated blood sampling from the inferior vena cava (IVC) at the juncture with the right adrenal vein (rt.AdV) [5]. The study included 44 patients with primary aldosteronism (PA) who had experienced adrenal venous sampling with adrenocorticotropic hormone (ACTH), resulting in a diagnosis of idiopathic hyperaldosteronism (IHA) (*n* = 24) and unilateral aldosterone-producing adenoma (APA) (*n* = 20; rt.APA = 8, lt.APA = 12). After the regular blood sampling was completed, blood was also sampled from the IVC of the substitute rt.AdV. Then, diagnostic performance was compared with the general lateralized index (LI) and the modified LI using the substitute rt.AdV. According to the result of the study, the modified LI of the rt.APA (0.4 ± 0.4) was lower than the IHA (1.4 ± 0.7) and the lt.APA (3.5 ± 2.0); the modified LI of the lt.APA was higher than the IHA and rt.APA. With this in mind, a more significant number of cases is certainly needed for future research; however, the proposed modified LI using blood sampled from the IVC at the junction of the right AdV may be considered an ancillary method.

Lee et al. compared chuna manual therapy (CMT) diagnostic methods, palpation, X-ray, and artificial intelligence (AI) programs in the lumbar spine and explored the clinical applicability of the CMT-AI program [6]. A total of 100 participants were recruited, and diagnostic modalities were analyzed using manual diagnosis, X-ray image-based diagnosis (XRD), and XRD using a CMT-AI program. The diagnostic results between the groups revealed that the gold standard was the highest for XRD using the AI program; therefore, it can be assumed that the clinical applicability of the CMT-AI program may be increased. Nonetheless, it is crucial to excessively increase the sample number to test the reliability and validity of the proposed CMT-AI program by using a test set in addition to utilizing various AI algorithms.

In the study by D’Attilio et al., a 3D Enlow’s counterpart analysis was performed on cone-beam computed tomography (CBCT) images [7]. For this study, skeletal Class I (ANB = 2° ± 2°) CBCT images of the 18 participants who had no history of orthodontic treatments were acquired. A 2D Enlow’s counterpart analysis was completed for each participant on lateral cephalograms extracted from the CBCT images. Overall, it is noteworthy that the proposed landmarks effectively identified the craniofacial structures in the 3D images.

Ali et al. carried out a national survey to analyze clinical arch forms and dimensions in the Iraqi population [8]. The results showed that the males’ linear measurements were more significant based on collecting the 1005 pre-treatment mandibular models. Notably, the arch forms were distributed in ovoid (47%), tapered (36.2%), and square (16.8%) forms. In conclusion, the ovoid arch form was predominant in classes I and III, while the tapered arch form was found to be dominant in class II. Hence, ovoid and tapered archwires should work well for most Iraqi patients.

Kim et al. performed a performed a pilot observational clinical study to compare metabolites and gut microbes between patients with ulcerative colitis (UC) and healthy individuals, seeking the potential for further investigation in developing an integrative medicine approach [9]. Blood and stool samples were collected from the participants; then, metabolite and gut microbial studies were performed. The study indicated that the metabolites and gut microbes significantly differed between the UC and HC groups and were mainly related to energy metabolism and inflammation processes. The results were positive; however, future studies on this topic must implement a well-designed randomized controlled trial with specified integrative medicine modalities to seek more concrete results, validating safety and effectiveness.

A study by Cho et al. aimed to analyze the performance of diagnostic musculoskeletal ultrasound training for Korean medicine students [10]. Students learned how to diagnose carpal tunnel syndrome, scan the volar wrist, and examine tendons, arteries, nerves, etc. There were two student groups: one group had eight weeks of training with a mock Objective Structured Clinical Examination (OSCE), and the other group had three weeks of training with flipped learning. A total of 60 students completed the questionnaire, and the analysis was performed using OSCE scores evaluated with a pre-validated checklist. The OSCE score was higher in the group that practiced for eight weeks; in addition, satisfaction with the ultrasound training was high (4.5 ± 0.60). Further ultrasound training on other anatomical areas and guided interventions could have considerable benefits if taught to traditional medicine students; however, traditional medicine students would benefit from practicing integrative medicine approaches, combining conventional medicine and complementary and alternative medicine (CAM) modalities for the betterment of patient care.

Two review papers were published in this Special Issue. One article highlighted that the diagnostic applications of wrist-worn wearables have proven their usefulness in diagnosing and monitoring diseases such as cardiovascular diseases, neurological diseases, liver diseases, psychological illnesses, and so on [11]. The study further illustrated that there are numerous machine learning techniques that can be applied for the analysis of wearable data for the early detection of diseases. This article has shown potential for the rapid growth of the market of smart wearable devices. The second review article used a systematic review protocol. The objective of the systematic review was to investigate diagnostic tools for the detection of Mizaj, as known as the primary diagnostic principle, in Persian medicine [12]. According to the review, whole-body Miraj was diagnosed in 37 studies using different questionnaires and 10 using expert panels. Moreover, six articles were used to examine the Mizaj of organs. Only four of these questionnaires reported reliability and validity. The study indicated that no sufficient data were available to assess the reliability and validity of the questionnaires.

Based on the published articles, the field of IM has earned substantial interest. Notably, the number of publications regarding IM has doubled from 2012 to 2021 [13]. As IM shows prospects of a bright future, there are a few important matters to mention. First, there still is a misconception that conventional healthcare professionals, especially physicians and nurses, believe that combining conventional approaches and complementary therapies shows no effects without seeking adequate evidence or considering trying. Second, well-designed clinical trials are essential, including a large number of patients. Since complementary and alternative medicine is heavily used in East Asia, trials in other parts of the world would be extremely valuable. Third, the era of AI medicine has arrived and has been extremely fruitful in the research community [14]. Though utilization of AI techniques has not been widespread among CAM practitioners, research has been conducted regarding AI techniques in CAM modalities [15]. With all that said, integrating AI or digital health technologies with conventional medicine and complementary therapies would also be extremely helpful in developing various integrative medicine modalities for delivering comprehensive patient care.

On a final note, the publications in this Special Issue have shown excellent potential for utilizing IM in the near future. The articles certainly provide great insights to the medical community; however, it is vital to reiterate that finding substantial evidence of the efficacy and safety of IM is still needed to optimize diagnostic validity and utility. Finally, the Guest Editor would like to express profound gratitude to all the authors who contributed to the Special Issue and the editorial staff, especially the assistant editor Gracie Zhang, who provided the utmost support.
